# In vitro propagation and DNA barcode analysis of the endangered *Silene schimperiana* in Saint Katherine protectorate

**DOI:** 10.1186/s43141-020-00052-8

**Published:** 2020-08-10

**Authors:** Heba El-Sayed Ghareb, Shafik Darwish Ibrahim, Ghada Abd El-Moneim Hegazi

**Affiliations:** 1 Tissue Culture Unit, Department of Genetic Resources, Desert Research Center, 1 Mathaf El-Matareya Street, Cairo, El-Matareya 11357 Egypt; 2 Agricultural Genetic Engineering Institute, Agricultural Research Center, Giza, Egypt

**Keywords:** Caryophyllaceae, Micropropagation, DNA barcoding, Southern Sinai, Egypt

## Abstract

**Background:**

Anthropogenic activity, climate change, pollution, and exploitation of natural resources are some reasons that cause threatening of plant diversity. *Silene schimperiana* is an endangered plant species in Egypt and is endemic to the high mountain of Saint Katherine Protected Area in southern Sinai. The purpose of the study was the ex situ conservation of *Silene schimperiana* through in vitro propagation and DNA barcode analysis.

**Results:**

To develop an efficient ex situ conservation program of the plant, in vitro propagation protocol has been achieved from shoot tip and stem nodal segment explants of in vitro germinated seedlings. Explants were established in vitro on Murashige and Skoog (MS) medium supplemented with 2.89 μM gibberellic acid (GA_3_)_,_ 1.08 μM α-naphthaleneacetic acid (NAA), and 1.16 μM kinetin (Kin). The highest number of axillary shoots (9.27) was obtained when they were transferred to MS medium supplemented with 4.48 μM 6-benzyl adenine (BA). Hundred percent of multiple axillary shoots were rooted on quarter-strength MS medium supplemented with 4.92 μM indole-3-butyric acid (IBA) and 10.75 μM NAA. Rooted plants were transferred to pots containing a soil-peat mixture (1: 2 v/v) and successfully acclimatized in the greenhouse. Plant identification is a crucial aspect to understand and conserve plant diversity from extinction. DNA barcode analysis of *Silene schimperiana* was carried out using two chloroplast DNA markers (cpDNA): 1,5-bisphosphate carboxylase/oxygenase large subunit (*rbcL*) and RNA polymerase subunit (*rpoC1*) and a nuclear ribosome DNA marker (ncDNA), internal transcribed spacer (*ITS*). Phylogenetic analysis revealed a successful identification of *Silene schimperiana* on the species and genus levels and supported the inclusion of *Silene schimperiana* in genus *Silene.*

**Conclusions:**

In this study, a relevant in vitro propagation method was established to facilitate the recovery of *Silene schimperiana*, in addition to DNA barcoding of the plant as a tool for effective management and conservation of plant genetic resources.

## Background

Saint Katherine Protectorate is one of the largest protected areas in Egypt with the highest mountains. It supports surprising biodiversity and a high proportion of endemic and rare plants. The flora of the mountains differs from the other areas, due to its unique geological, morphological, and climatic aspects [1]. The protection and conservation of endemic plant species is a worldwide need; especially under the threat of climate change. Endemic species are the most defenseless, because of their unique evolutionary history and low population estimate [2].

*Silene schimperiana* Boiss. is a perennial herb, belonging to the largest genus from family Caryophyllaceae. Its Arabic name is Wesbi or Losseiq [3]. The plant is a hemicryptophyte, growing on rocky wadi beds. It is potentially an ornamental plant with attractive white flowers [4] and has economic importance in grazing processes as a pastoral plant [5].

*Silene schimperiana* is endangered in Egypt and is endemic to the high mountain of Saint Katherine Protected Area in southern Sinai. The plant populations are severely fragmented with a continuing decline in habitat quality [5]. Drought is the major threat affecting the distribution of *Silene schimperiana*, in addition to climate change. The plant has low viability due to destructive overgrazing, causing loss of reproductive organs. With drought, the effect of overgrazing is more harmful and the wild populations of this species could be in extreme danger in the near future [5]. Ex situ conservation by in vitro propagation and molecular identification are important actions needed to conserve the plant [5].

In vitro propagation of other species of *Silene* have been achieved previously, such as *S. leucophylla* [6], *S. fetissovii*, *S. obovata*, *S. sussamyrica* and *S. ladyginae* [7], *Silene cretacea* [8], *Silene bolanthoides* [9] and *Silene fabaria* subsp. *domokina* [10].

Identification of rare and endemic plant species is an important base for evolutionary and phytogeographic studies as well as for the determination of conservation priorities [11].

DNA barcoding is a molecular marker-based technique, in which short fragments of DNA from a standardized genome position are utilized. This technique identifies plant species more definite than the traditional taxonomic tools, with unrecognizable plant parts and without requiring taxonomic experience [12, 13]. DNA barcoding has applications in biodiversity monitoring, conservation impact assessment, forensic botany, monitoring illegal trading, etc. Also, DNA is stable and is found in all tissues; therefore, DNA barcoding of medicinal plants can be used for identifying powdered or processed plant materials [14].

Studying the molecular phylogeny of plants depends mainly on the sequencing of the chloroplast genome, because of its simple and stable genetic structure. Universal primers are used for these target sequences amplification, such as 1,5-bisphosphate carboxylase/oxygenase large subunit (*rbcL*) and RNA polymerase subunit (*rpoC1*) have been heavily relied upon for the development of markers for plant DNA barcoding [15]. The Consortium for the Barcode of Life (CBOL) [16] plant working group evaluated the efficacy of the chloroplast DNA marker (cpDNA), *rbcL* among the recommended markers. The *rbcL* gene is the most published plastid barcode due to its high amplification success rate and is considered a core DNA barcoding marker for the determination of plant diversity [13, 17, 18]. Besides, the nuclear ribosome DNA marker (ncDNA), internal transcribed spacer (*ITS*) and cpDNA, *rpoC1* were frequently evaluated as plant barcodes [12, 19]. DNA barcoding markers are universal and the choice of the correct loci is challenging [20].

To date, there has been no published researches on the in vitro culture or DNA barcoding of *Silene schimperiana* and therefore, the aim of the present study was the ex situ conservation of *Silene schimperiana* as an endangered plant, endemic to Saint Katherine, through in vitro propagation and identification by DNA barcode analysis using two cpDNA genes: *rpoC1* and *rbcL* and a ncDNA gene, *ITS*.

## Methods

### Plant material

**Fig. 1:**
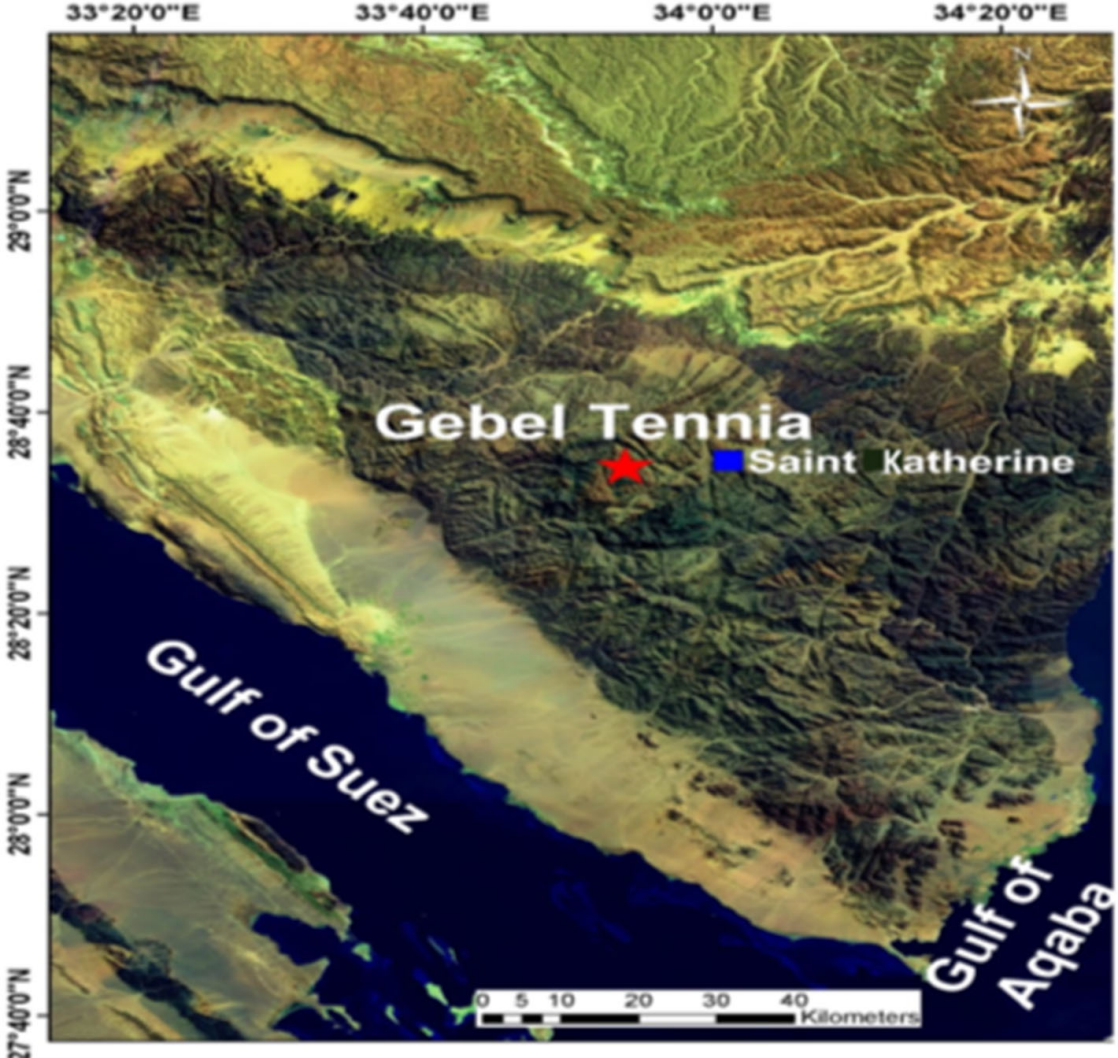
A map showing the location of Gebel Tennia, the collection site of *Silene schimperiana*

**Fig. 2:**
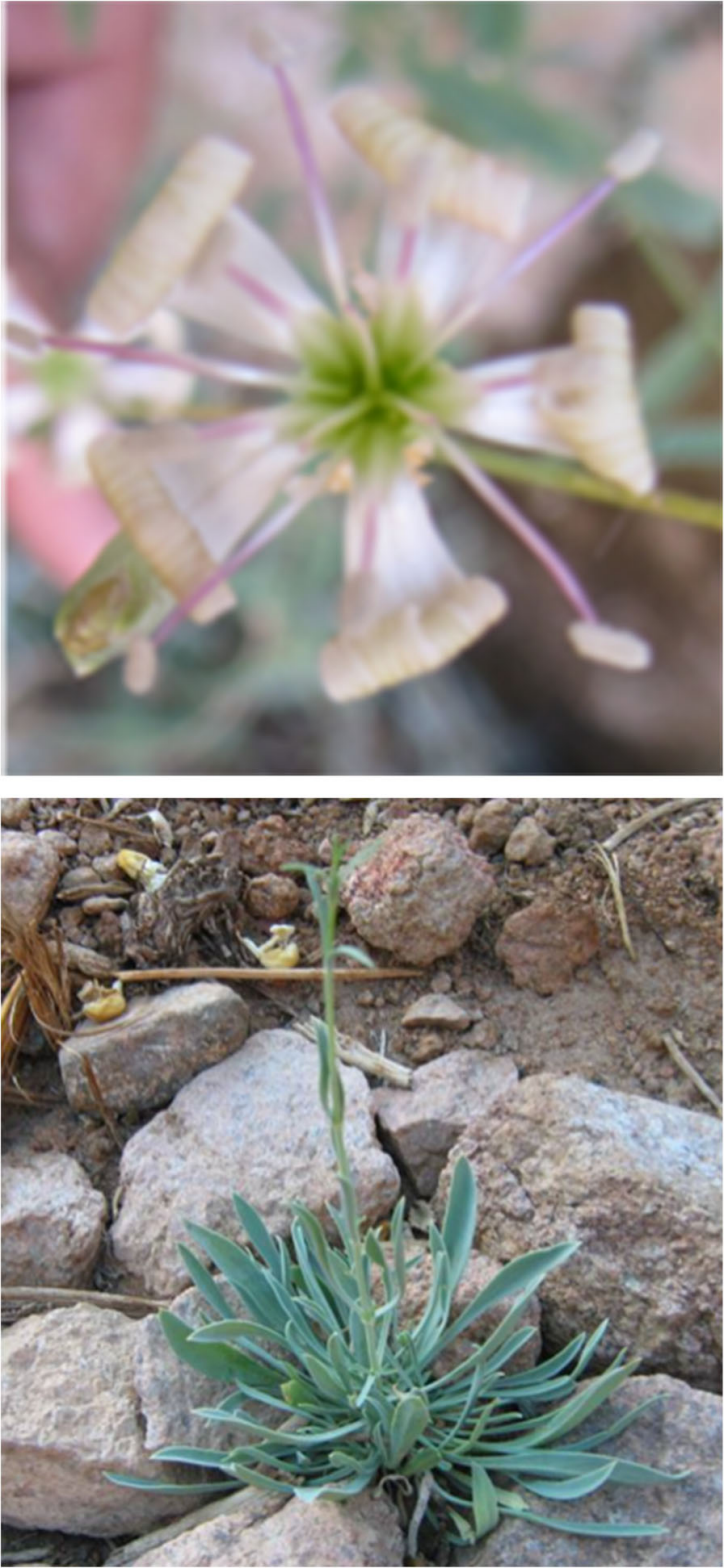
*Silene schimperiana* plant growing naturally in Gebel Tennia, Saint Katherine, Southern Sinai, with a close up view of a flower

Leaf specimens and seeds of *Silene schimperiana* were collected from shrubs grown in Gebel Tennia, Saint Katherine, South of Sinai E: 33.90090 N: 28.57364 Alt: 1810 (Figs. 1 and 2). Identification of plant was carried out by Dr. Omran Ghaly, Head of Plant Taxonomy Unit, Desert Research Center, Egypt, given the voucher number CAIH-1003-R and voucher specimens were deposited in the Herbarium of Desert Research Center (CAIH).

### In vitro experiments

#### In vitro germination

Collected seeds of *Silene schimperiana* were washed with running tap water and detergent, then surface sterilized by soaking in commercial bleach containing sodium hypochlorite (5.25%) under laminar airflow cabinet (Holten LaminAir HVR 2448, USA), at different concentrations (0.5, 1.0, and 1.5% sodium hypochlorite solution). After 15 min, seeds were taken out and thoroughly washed thrice with sterilized distilled water. Seeds were cultured on half-strength Murashige and Skoog (1/2 MS) [21] medium (Duchefa, Haarlem, the Netherlands) supplemented with 3% (w/v) sucrose and 5.77 μM gibberellic acid (GA_3_; Sigma Cell Culture, min. 90%, St. Louis, USA) (0.45 μm filter sterilized) for germination. The pH was adjusted to 5.7 ± 0.1, then medium was gelled with 0.3% (w/v) phytagel (Duchefa, Haarlem, the Netherlands) before autoclaving at a pressure of 1.06 kg/cm, and 121 °C for 15 min (Harvey Sterilemax autoclave, Thermo Scientific, USA). Cultures were incubated under cool fluorescent tubes at day-night regime of 16-h photoperiod with the light intensity of 2500-3000 lux (F140t9d/38, Toshiba) at a constant temperature of 25 ± 2 °C and 60-70% relative humidity. The produced seedlings were cut into shoot tips and stem nodal segments and were used as explants.

#### In vitro propagation

##### Establishment stage

Shoot tips and stem nodal segment explants of seedlings were transferred to MS medium supplemented with 3% (w/v) sucrose and 0.3% (w/v) phytagel and 2.89 μM GA_3_, 1.08 μM α-naphthalene acetic acid (NAA) and different concentrations of 6-benzyl adenine (BA) of 1.11, 2.22, and 4.48 μM or kinetin (Kin) of 1.16, 2.33, and 4.65 μM (Sigma Cell Culture, min. 90%, St. Louis, USA). MS medium without plant growth regulators (PGRs) served as a control. The pH of the medium was adjusted to 5.7 ± 0.1 and autoclaved at a pressure of 1.06 kg/cm and 121 °C for 15 min. Cultures were incubated at 25 ± 1 °C at a photoperiod of 16/8 h light/darkness under cool white fluorescent tubes of 2500-3000 lux. The survival and growth percentage (%), the mean number and length (cm) of axillary shoots/explant were recorded after 3 weeks of culture.

##### Multiplication stage

The in vitro produced axillary shoots were transferred to MS medium supplemented with 3% (w/v) sucrose and 0.3% (w/v) phytagel and different concentrations of cytokinins (Sigma Cell Culture, min. 90%, St. Louis, USA); BA (2.22, 4.48, and 8.97 μM), N6-(2-isopentenyl) adenine (2iP; 2.44, 4.83, and 9.65 μM); or thidiazuron (TDZ; 2.27, 4.55, and 9.1 μM), individually. MS medium without PGRs served as a control. The pH of the medium was adjusted, autoclaved, and cultures were incubated as mentioned in the shoot induction stage. The number and length (cm) of axillary shoots/explant were recorded after 3 weeks of culture. Subculturing was done every 3 weeks.

##### Rooting and acclimatization stages

The multiplied axillary shoot clusters were transferred for rooting on quarter-strength MS medium supplemented with different combinations of indole-3-butyric acid (IBA; Sigma Cell Culture, min. 90%, St. Louis, USA) at 4.92 and 9.85 μM and NAA at 5.38 and 10.75 μM. Quarter-strength MS medium without PGRs served as a control for rooting induction. The pH was adjusted, media autoclaved, and cultures were incubated as mentioned in the previous stages. The percentage of rooted shoots (rooting %) and the number and length (cm) of roots/explant were scored after 4 weeks of culture on the rooting medium.

The rooted plantlets were removed from the nutrient medium and washed thoroughly with distilled water to eliminate the residues of the medium, then were transferred to plastic pots in a mixture of sand and peat (1: 2 v/v) (Peat moos, PROMIX®). The pots were covered by translucent polythene plastic bags to maintain high humidity and prevent the dissection of the newly transferred plantlets. Transplants were irrigated and the plastic bags were pored (one pore/5 days for 5 weeks) to decrease the humidity and acclimatize the plants to the external atmosphere gradually. After 6 weeks, the plastic bags were removed completely and the plants allowed to grow under open conditions.

#### Experimental design and statistical analysis

In vitro experiments were subjected to the completely randomized design. At least ten replicates were cultured for each treatment and the experiments were repeated twice. One-way analysis of variance (ANOVA) was used to evaluate significant differences between the mean values of different treatments, using Duncan’s Multiple Range Test [22] as modified by Snedecor and Cochrane [23]. The differences between means were compared at *p* < 0.05.

### DNA barcode analysis

#### Extraction and purification of DNA

*Silene schimperiana* leaf specimens were collected and ground to a fine powder in liquid nitrogen using a sterile mortar and pestle. For DNA extraction and purification, DNeasy Plant Kit (Qiagen, Germany) was used. The concentration and quality of extracted DNA were estimated by running on 1% agarose gel electrophoresis, using a DNA size marker (Lambda DNA *Hind III* digest Phi X 174/HaeIII digest).

#### PCR and gene sequencing

The PCR reaction was carried out as reported by Ibrahim et al. [24] in a total volume of 50 μL PCR master mixture consisted of the following: 1x buffer (Promega, USA), 15 mM MgCl_2_, 0.2 mM dNTPs (Promega, USA), 20 pcoml of each primer (Invitrogen, USA), 1 u of Taq DNA polymerase (GoTaq, Promega, USA), 40 ng DNA and ultra-pure water to the final volume. DNA barcoding was performed with the ncDNA *ITS* gene and two cpDNA genes, *rpoC1* and *rbcL*. For PCR amplification and sequencing of *rpoC1*, *rbcL*, and *ITS*, the following primer pairs were used: *ITS-F* (5′-CCT TAT CAT TTA GAG GAA GGA G-3′), *ITS-R* (5′-TCC TCC GCT TAT TGA TAT GC-3′); *rpoC1-F* (5′-GGC AAA GAG GGA AGA TTT CG-3′), *rpoC1-R* (5′-CCA TAA GCA TAT CTT GAG TTG G-3′); and *rbcL-*F (5′-ATG TCA CCA CAA ACA GAA AC-3′), *rbcL-R* (5′-TCG CAT GTA CCT GCA GTA GC-3′). The average amplicon sizes/bp were 722, 520, and 693 for *ITS*, *rpoC1*, and *rbcL*, respectively.

The PCR was carried out with a Perkin-Elmer/GeneAmp® PCR System 9700 (PE Applied Biosystems, USA) programmed to fulfill 40 cycles after an initial denaturation cycle for 5 min at 94 °C. Each cycle consisted of a denaturation step at 94 °C for 30 s, an annealing step at 50 °C for 30 s, and an elongation step at 72 °C for 30 s. The primer extension segment was extended to 7 min at 72 °C in the final cycle.

The amplification products were determined by electrophoresis in a 1.5% agarose gel using ethidium bromide (0.5 ug/ml) in 1X Tris borate Edita (TBE) buffer at 95 volts. For PCR product size determination, a 100 bp DNA ladder (Promega, USA) was used as a molecular size standard. Gel images were visualized using a UV transilluminator and photographed using a Gel Documentation System (BIO-RAD 2000, USA).

Purification of PCR products was carried out by a QIAquick PCR Purification Kit (Qiagen, USA). The PCR product was sequenced using the dideoxynucleotide chain termination method with a DNA sequencer (ABI 3730XL, Applied Biosystems) (Microgen, Korea) and a BigDye Terminator version 3.1 Cycle Sequencing RR-100 Kit (Applied Biosystems, USA) according to the protocol supplied by the manufacturer.

#### Assignment of species

DNA barcoding of *Silene schimperiana* was carried out using the Basic Local Alignment Tool (BLAST) available on the National Center of Biotechnology Information (NCBI) website. All sequences were submitted to GenBank, USA. It provided MK628682, MK628685, and MK628687 accession numbers for the nucleotide sequences.

BLAST searches were applied to all produced sequences using the online databases (DDBJ/EMBL/GenBank), analyzed using BLASTN 2.9.0 program (http://www.ncbi.nlm.nih.gov/BLAST), and aligned using Align Sequences Nucleotide BLAST. The identification of species was considered successful when the highest similarity percentage included a single species scored more than 97% [20]. Phylogenetic analysis was conducted using MAFFTv6.864, http://www.genome.jp/tools-bin/mafft, and phylogenetic trees were generated.

## Results

### In vitro propagation of *Silene schimperiana*

#### Establishment of in vitro culture

Eighty percent of *Silene schimperiana* seeds was survived and germinated when sterilized with 1% of sodium hypochlorite solution for 15 min and planted on 1/2 MS medium supplemented with 5.77 μM GA_3_. Shoot tips and stem nodal segments were excised from 1-month-old seedlings and transferred to the different establishment media to achieve optimization of the in vitro culture (Table 1). MS medium supplemented with 2.89 μM GA_3_, 1.08 μM NAA, and different concentrations of either BA or Kin was examined. All treatments gave 100% of both survived explants and growth formation for both shoot tip and stem nodal segment explants.

**Table 1:** Effect of Murashige and Skoog (MS) medium supplemented with 2.89 μM gibberellic acid (GA_3_) and 1.08 μM α-naphthaleneacetic acid (NAA) in addition to 6-benzyl adenine (BA) or kinetin (Kin) on the in vitro establishment of *Silene schimperiana* shoot tip and stem nodal segment explants. All treatments gave 100% of both survived explants and growth formation

Cytokinin conc. (μM)		Shoot tip		Stem nodal segment	
BA	Kin	Mean no. of axillary shoots/explant	Mean length of axillary shoots (cm)	Mean no. of axillary shoots/explant	Mean length of axillary shoots (cm)
**0.00**	**0.00**	2.0^b^	1.30^b^	2.3^b^	1.39^b^
**1.11**	**0.00**	2.7^ab^	2.00^ab^	2.9^b^	2.28^ab^
**2.22**	**0.00**	2.6^ab^	1.70^b^	2.9^b^	2.06^ab^
**4.48**	**0.00**	2.0^b^	1.43^b^	2.3^b^	1.50^b^
**0.00**	**1.16**	3.7^a^	2.91^a^	4.3^a^	3.00^a^
**0.00**	**2.33**	3.2^ab^	2.10^ab^	4.0^a^	2.33^ab^
**0.00**	**4.65**	2.2^b^	1.70^b^	2.4^b^	2.10^ab^

Means in the same column with different letters are statistically significantly different at *p* ≤ 0.05

**Fig. 3:**
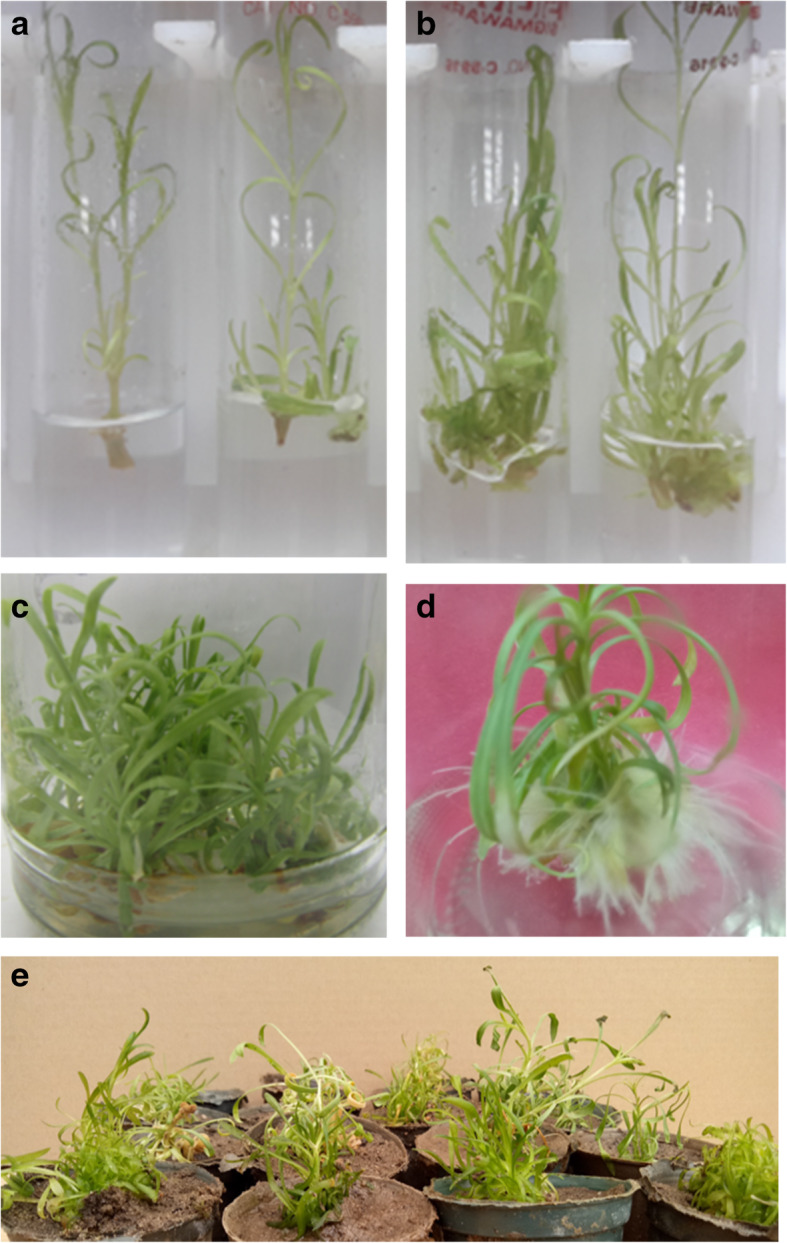
In vitro propagation of *Silene schimperiana*; (**a**) in vitro establishment of shoot tip, (**b**) in vitro establishment of stem nodal segment, (**c**) multiplication of axillary shoots, (**d**) rooted plantlet, and (**e**) acclimatization of transplants in the greenhouse

Concerning shoot tip explant, the mean number and length of axillary shoots per explant ranged between 2.0 and 3.7 shoots with a length of 1.3 and 2.91 cm, respectively. The maximum number and length of axillary shoots were achieved on the medium supplemented with 1.16 μM Kin (Fig. 3a). Increasing the concentration of Kin led to a decrease in the number and length of axillary shoots. The same trend was observed with BA, as the highest mean number and length of axillary shoots per explant was recorded at the lowest concentration (1.11 μM) and it decreased by increasing BA concentration (Table 1).

Concerning the stem nodal segment explant, as shown in Table 1, it recorded higher numbers and lengths of axillary shoots per explant on all tested media comparing to shoot tip explant. The mean number and length of axillary shoots per explant ranged between 2.3 and 4.3 shoots and 1.39 and 3.0 cm, respectively. The highest number and length of axillary shoots were achieved on the same optimum medium for shoot tip explant that supplemented with 1.16 μM Kin (Fig. 3b), and they also decreased with increasing the concentration of Kin. On the other hand, BA gave lower values of the mean number and length of axillary shoots compared to the same concentrations of Kin. Concerning the control treatment without cytokinin, it gave the minimum response for both explants.

In conclusion, for the in vitro establishment of *Silene schimperiana*, MS medium supplemented with 2.89 μM GA_3_, 1.08 μM NAA, and 1.16 μM Kin was the best medium for stem nodal segment and shoot tip explants. Comparing the two tested explants, it was found that the stem nodal segment had a higher shoot induction capacity than the shoot tip.

#### Multiplication of axillary shoots

Multiplication of the in vitro established axillary shoots was achieved as shown in Table 2. All tested treatments enhanced multiple shoots production, which ranged between 3.27 and 9.27 axillary shoots per explant. The significantly highest mean number of axillary shoots per explant was recorded on MS medium supplemented with 4.48 μM BA, followed by 8.97 μM BA. Concerning the other tested cytokinins, 2iP ranked next as it produced a higher number of axillary shoots than the same concentrations of TDZ. The number of axillary shoots increased by increasing the concentration of 2iP.

**Table 2:** Effect of Murashige and Skoog (MS) medium supplemented with 6-benzyl adenine (BA), N6-(2-isopentenyl) adenine (2iP), or thidiazuron (TDZ) on the in vitro multiplication of *Silene schimperiana* axillary shoots

Cytokinin conc. (μM)			Mean no. of axillary shoots/explant	Mean length of axillary shoots (cm)
BA	2iP	TDZ		
**0.00**	**0.00**	**0.00**	3.27^d^	1.4^a^
**2.22**	**0.00**	**0.00**	5.45^bc^	1.3^a^
**4.48**	**0.00**	**0.00**	9.27^a^	1.9^a^
**8.97**	**0.00**	**0.00**	7.27^b^	1.8^a^
**0.00**	**2.44**	**0.00**	4.36^cd^	2.1^a^
**0.00**	**4.83**	**0.00**	4.73^cd^	1.8^a^
**0.00**	**9.65**	**0.00**	5.64^bc^	1.8^a^
**0.00**	**0.00**	**2.27**	4.00^cd^	1.5^a^
**0.00**	**0.00**	**4.55**	3.64^cd^	1.9^a^
**0.00**	**0.00**	**9.10**	5.18^cd^	1.4^a^

Means in the same column with different letters are statistically significantly different at *p* ≤ 0.05

Regarding the mean length of axillary shoots, it was insignificantly different for all tested treatments and the maximum length of 2.1 cm was recorded with 2.44 μM 2iP. The medium without cytokinin recorded the lowest mean number and length of axillary shoots. In this experiment, 2iP (at 4.83 and 9.65 μM) and TDZ (at all tested concentrations) caused the vitrification of axillary shoots.

In conclusion, BA was the most effective tested cytokinin for the multiplication of non-vitreous axillary shoots, compared to both 2iP and TDZ. While TDZ gave the minimum response. The significantly highest mean number of axillary shoots per explant was recorded on MS medium supplemented with 4.48 μM BA (Fig. 3c).

#### Rooting of axillary shoots and acclimatization of plantlets

Combinations between IBA and NAA were tested for rooting of axillary shoots of *Silene schimperiana* and gave promising results as presented in Table 3.

**Table 3:** Effect of quarter-strength Murashige and Skoog (¼ MS) medium supplemented with indole-3-butyric acid (IBA) and α-naphthaleneacetic acid (NAA) at different combinations on the rooting of *Silene schimperiana* multiple shoot clusters

Auxin conc. (μM)		Rooting (%)	Mean no. of roots/explant	Mean length of roots (cm)	Mean length of axillary shoots (cm)
IBA conc. (μM)	NAA conc. (μM)				
**0.00**	**0.00**	33	16.88^c^	3.4^e^	5.0^c^
**4.92**	**5.38**	100	30.13^a^	6.0^b^	7.0^b^
**4.92**	**10.75**	100	34.50^a^	7.4^a^	8.5^a^
**9.85**	**5.38**	66	22.31^b^	5.2^c^	6.5^b^
**9.85**	**10.75**	66	18.65^bc^	4.2^d^	5.5^c^

Means in the same column with different letters are statistically significantly different at *p* ≤ 0.05

The rooting percentage reached 100% using 4.92 μM IBA in combination with either 5.38 or 10.75 μM NAA in quarter-strength MS medium. Also, the mean number and length of roots per explant and mean length of axillary shoots were maximum at the same concentrations. The medium without auxin recorded the lowest rooting percentage, mean number and length of roots, and mean length of axillary shoots. Regarding the highest number of roots, the concentration of 4.92 μM IBA in combination with 10.75 μM NAA is the optimum concentration for the rooting of *Silene schimperiana*, which produced 34.5 roots per explant with also the highest mean length of roots (7.4 cm) and axillary shoots (8.5 cm) (Fig. 3d), followed by the same concentration of IBA in combination with 5.38 μM NAA (30.13 roots per explant of 6.0 cm roots and 7.0 cm axillary shoots).

At the end of the rooting stage, the rooted plantlets were successfully acclimatized to ex vitro conditions into pots containing a soil-peat mixture (1: 2 v/v) and grown in greenhouse conditions with an approximately 75% survival rate (Fig. 3e).

### Identification of *Silene schimperiana* by DNA barcode analysis

**Fig. 4:**
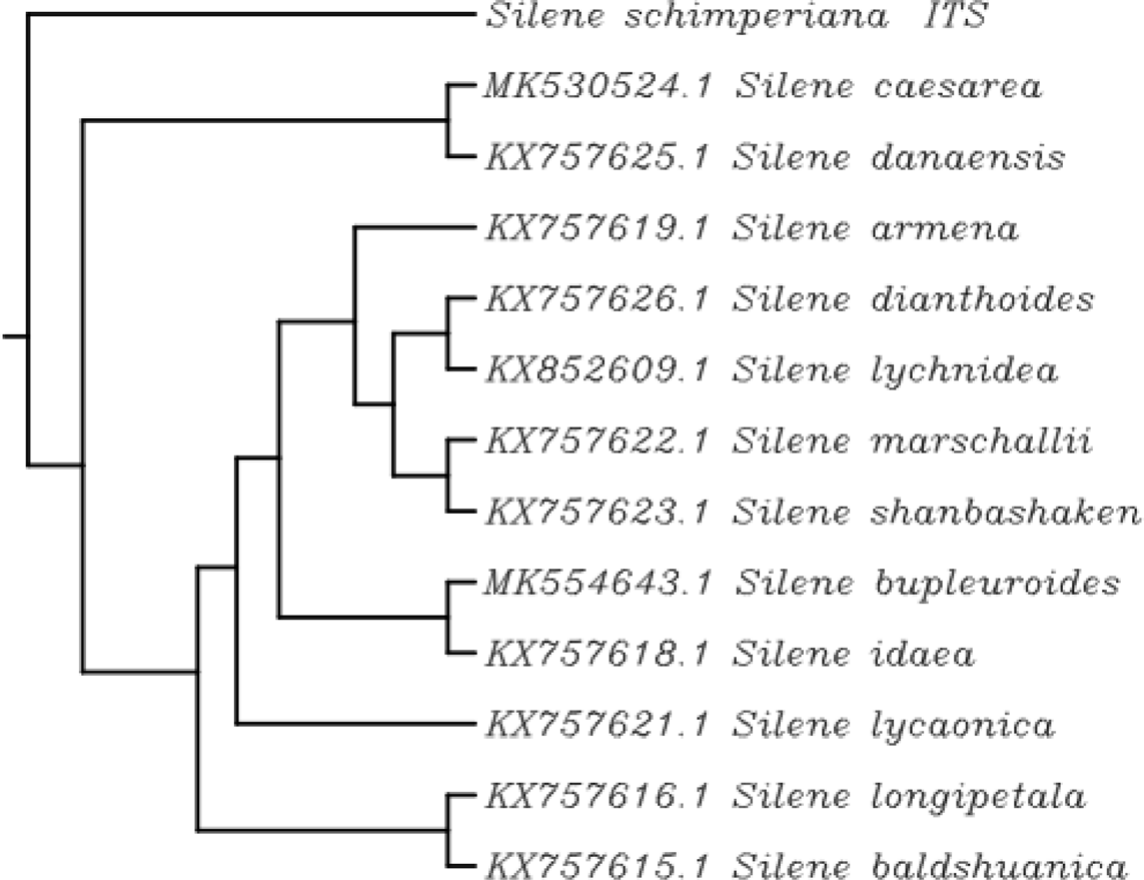
Phylogenetic tree of *Silene schimperiana* using the ncDNA: internal transcribed spacer (*ITS*)

**Fig. 5:**
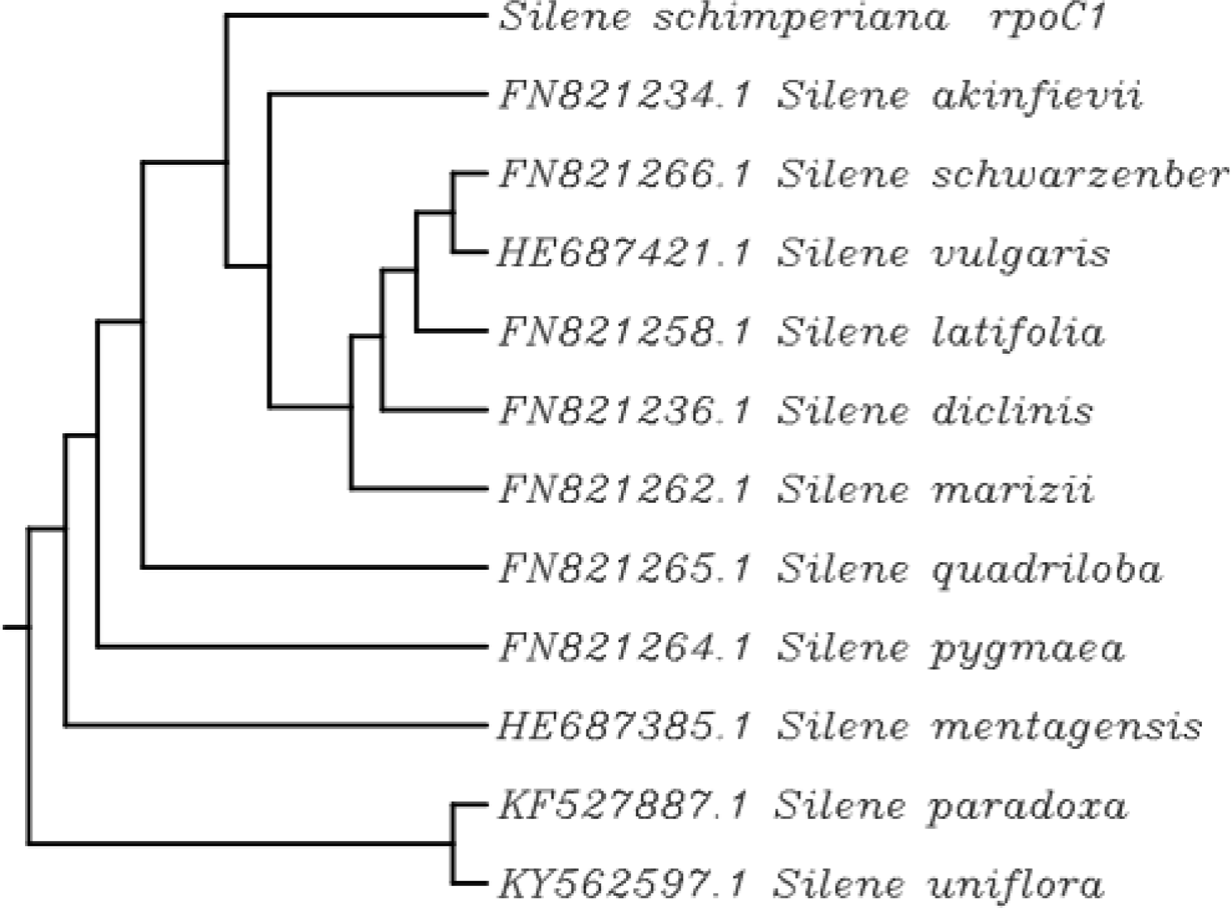
Phylogenetic tree of *Silene schimperiana* using the cpDNA marker: RNA polymerase subunit (*rpoC1*)

**Fig. 6:**
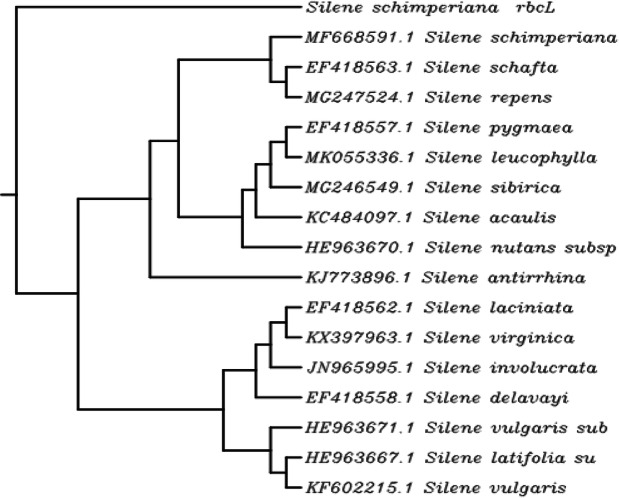
Phylogenetic tree of *Silene schimperiana* using the cpDNA marker: 1,5-bisphosphate carboxylase/oxygenase large subunit (*rbcL*)

**Fig. 7:**
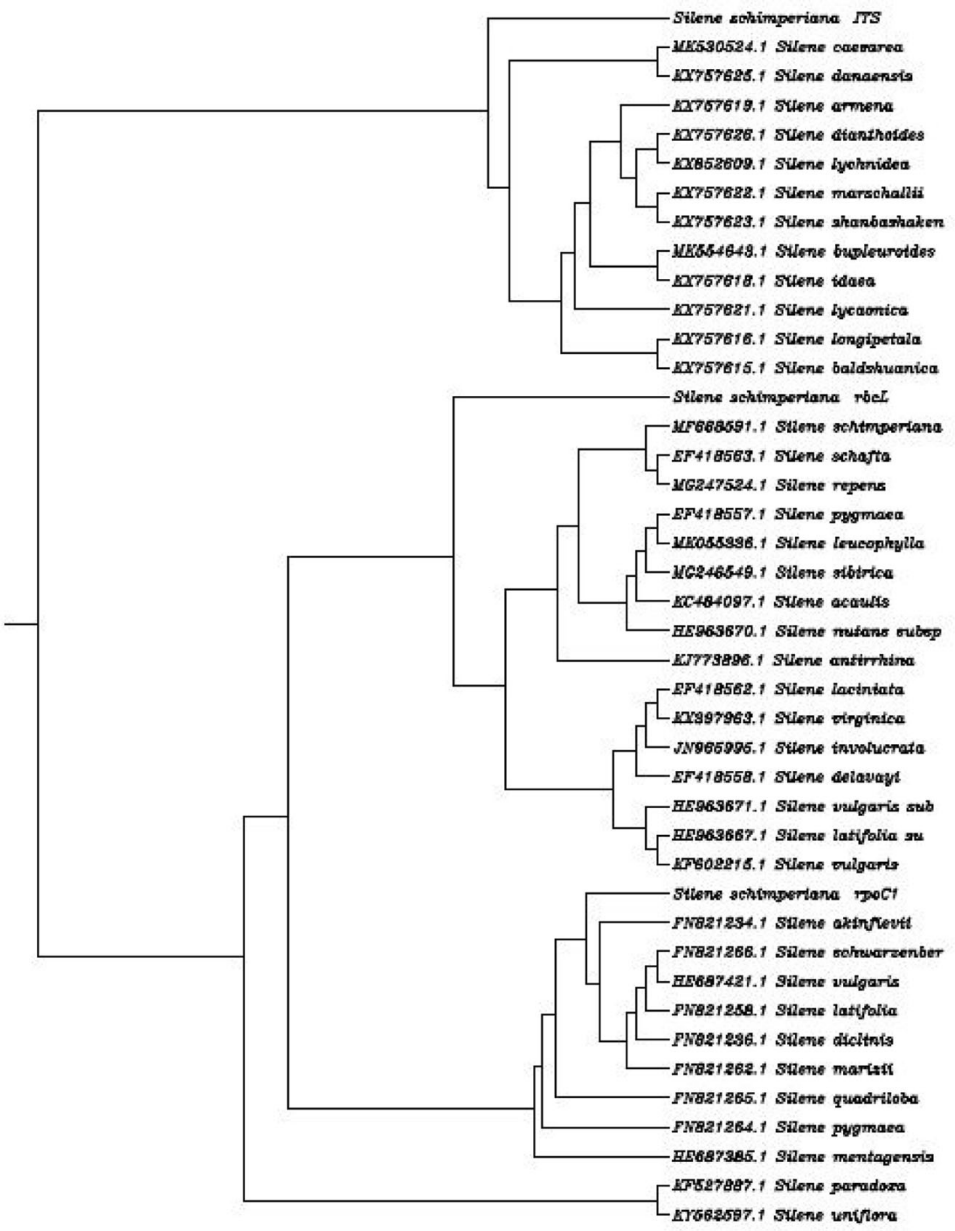
Combined phylogenetic tree of *Silene schimperiana* using the three markers: internal transcribed spacer (*ITS*), RNA polymerase subunit (*rpoC1*), and 1,5-bisphosphate carboxylase/oxygenase large subunit (*rbcL*)

For the identification and classification of the rare endemic *Silene schimperiana* plant for conservation, DNA barcoding was carried out. The results of BLAST matching and phylogenic tree analysis of *Silene schimperiana* are shown in Tables 4, 5, and 6 and Figs. 4, 5, 6, and 7. The plant species of the highest percentages of similarity are represented. Newly generated sequences of the three markers: *ITS*, *rpoC1*, and *rbcL* were used as barcodes. The alignments of *ITS*, *rpoC1*, and *rbcL* sequences against GenBank accessions yielded a query coverage between 62 to 66%, 83 to 94%, and 74 to 80%, respectively (Tables 4, 5, and 6). Sequencing for *ITS*, *rpoC1*, and *rbcL* regions of *Silene schimperiana* resulted in 697, 495 and 668 bp sequences (affected length of the query), respectively. Sequence alignment analysis revealed 100% the genus *Silene* hits of 737-874, 651-880, and 922-972 bp length for *ITS*, *rpoC1*, and *rbcL* sequences, respectively.

**Table 4:** DNA barcode of internal transcribed spacer (*ITS*) downloaded from GenBank database including plant species with similarity percentage of more than 97.78%

Plant species	Accession no.	***E*** value	Query coverage (%)	Similarity (%)
*Silene caesarea*	MK530524.1	0.0	66	99.38
*Silene danaensis*	KX757625.1	0.0	66	99.17
*Silene armena*	KX757619.1	0.0	66	98.76
*Silene dianthoides*	KX757626.1	0.0	66	98.14
*Silene lychnidea*	KX852609.1	0.0	63	97.80
*Silene marschallii*	KX757622.1	0.0	66	97.93
*Silene shanbashakensis*	KX757623.1	0.0	64	97.85
*Silene bupleuroides*	MK554643.1	0.0	66	97.93
*Silene idaea*	KX75618.1	0.0	62	97.79
*Silene lycaonica*	KX757621.1	0.0	66	98.54
*Silene longipetala*	KX757616.1	0.0	66	98.14
*Silene baldshuanica*	KX757615.1	0.0	63	98.48

**Table 5:** DNA barcode of RNA polymerase subunit (*rpoC*1) downloaded from GenBank database including plant species with similarity percentage of more than 98.77%

Plant species	Accession no.	***E*** value	Query coverage (%)	Similarity (%)
*Silene akinfievii*	FN821234.1	0.0	90	99.15
*Silene schwarzenbergii*	FN821266.1	0.0	90	99.15
*Silene vulgaris*	HE687421.1	0.0	86	99.33
*Silene latifolia*	FN821258.1	0.0	83	99.31
*Silene diclinis*	FN821236.1	0.0	83	99.31
*Silene marizii*	FN821262.1	0.0	90	98.93
*Silene quadriloba*	FN821265.1	0.0	90	98.93
*Silene pygmaea*	FN821264.1	0.0	90	98.93
*Silene mentagensis*	HE687385.1	0.0	86	98.89
*Silene paradoxa*	KF527887.1	0.0	94	98.98
*Silene uniflora*	KY562597.1	0.0	94	98.78

**Table 6:** DNA barcode of 1,5-bisphosphate carboxylase/oxygenase large subunit (*rbcL*) downloaded from GenBank database including plant species with similarity percentage of more than 98.2%

Plant species	Accession no.	***E*** value	Query coverage (%)	Similarity (%)
*Silene schimperiana*	MF66859.1	0.0	80	98.21
*Silene schafta*	EF418563.1	0.0	77	98.89
*Silene repens*	MG247524.1	0.0	77	98.50
*Silene pygmaea*	EF418557.1	0.0	77	98.70
*Silene leucophylla*	MK055336.1	0.0	77	98.88
*Silene sibirica*	MG246549.1	0.0	77	98.31
*Silene acaulis*	KC484097.1	0.0	77	98.31
*Silene nutans* subsp. *nutans*	HE963670.1	0.0	77	98.69
*Silene antirrhina*	KJ773896.1	0.0	75	98.67
*Silene laciniata*	EF418562.1	0.0	77	98.52
*Silene virginica*	KX397963.1	0.0	74	99.03
*Silene involucrata*	JN965995.1	0.0	74	98.84
*Silene delavayi*	EF418558.1	0.0	77	98.33
*Silene vulgaris* subsp. *vulgaris*	HE963671.1	0.0	77	98.32
*Silene latifolia* subsp. *alba*	HE963667.1	0.0	77	98.32
*Silene vulgaris*	KF602215.1	0.0	76	98.48

The highest similarity percentages between *Silene schimperiana* and the other recorded *Silene* species reached 99.38% for *ITS* with *Silene caesarea*, 99.33% for *rpoC1* with *Silene vulgaris* and 99.03% for *rbcL* with *Silene virginica* (Tables 4, 5, and 6)*.* The *rbcL* sequence showed 98.21% similarity with the same species, *Silene schimperiana*. So, *Silene schimperiana* was successfully identified on both species and genus levels for the *rbcL* marker and the genus level for *ITS* and *rpoC1* markers*.*

The phylogenetic trees of the plant species with the highest similarity percentages have a fan shape (Figs. 4, 5, 6, and 7), showing clustering of closely related species together and scattering of relatively distantly related species. The combined phylogenetic analysis represented in Fig. 7 shows that the three tested markers support the inclusion of *Silene schimperiana* in the genus *Silene* and the most closely related species are *Silene caesarea*, *Silene schimperiana*, and *Silene akinfievii*.

## Discussion

### In vitro propagation of *Silene schimperiana*

Shoot tips and stem nodal segments were successfully established in vitro on MS medium supplemented with 2.89 μM GA_3_, 1.08 μM NAA and different concentrations of either BA or Kin. The main property of GA_3_ is the stimulation of cell division and elongation and the presence of auxin (NAA) in the medium enhances the action of GA_3_ [8, 25]. This was confirmed by Kritskaya et al. [8], who successfully micropropagated *Silene cretacea* by using GA_3_ and auxin in the medium in a combination with cytokinin.

Don Palmer and Keller [26] reported that explant source is one of the important factors for the successful establishment of in vitro culture. According to the results of the present study, when the two explant types were compared, it was found that the stem nodal segment had a higher shoot induction capacity than the shoot tip. Similarly, Çördük et al. [9] found that nodal explant was the most effective explant for shoot regeneration of *Silene bolanthoides*.

For the in vitro establishment of *Silene schimperiana*, MS medium supplemented with 2.89 μM GA_3_, 1.08 μM NAA, and 1.16 μM Kin was the best medium for stem nodal segment and shoot tip explants, respectively. Kinetin, together with the auxin (NAA), participates in the organogenesis process in plants. This is supported by Kritskaya et al. [8], who found that Kin was effective in the micropropagation of *Silene cretacea.* Also, it has been reported on other *Silene* species that cytokinin and auxin are required in combination in tissue culture media and are the best for the initial shoot induction phase [10].

Multiplication of axillary shoots was influenced by cytokinin type and concentration. The significantly highest mean number of axillary shoots was recorded with BA, and 2iP ranked next, followed by TDZ. In this experiment, 2iP and TDZ caused the vitrification of axillary shoots. Therefore, it is important to select a proper concentration of cytokinin, because high concentrations cause vitrification and somaclonal variations [27]. Cytokinins (BA, 2iP, and TDZ) had multifunction in the physiological processes and the development of the plant as cell division and enlargement and stimulation of protein synthesis and enzyme activity [28]. The most typical cytokinin function in the plant in vitro culture is the suppression of apical dominance and the stimulation of lateral buds, due to the differentiation of vascular tissue between the axillary buds and vascular bundles of the main stem [8, 29].

The optimum medium for multiplication of axillary shoots was MS medium supplemented with 4.48 μM BA. It is clear from the results that the proper choice of cytokinin is one of the most important factors affecting the multiplication of axillary shoots. In the present study, BA was more effective in the production of non-vitreous multiple axillary shoots than 2iP and TDZ. The high efficiency of BA could be contributed to that it considered the most stable among the tested cytokinins, because of the stability of the aromatic side-chain substituted at N6 that is higher than the isoprenoid chain of 2iP. Also, some of the conjugated forms of BA are produced during BA metabolism, extending its action [30, 31]. Furthermore, BA is not a suitable substrate of cytokinin oxidase enzyme, which is responsible for the endogenous cytokinin balance [31]. Moreover, it is worth mentioning that among the various cytokinins, BA is the most effective, preferable, and cheapest cytokinin used for in vitro shoot multiplication [10]. Regarding TDZ, its minimum effect in comparison with BA and 2iP may be contributed to that in some systems, the synergistic effect of TDZ with other cytokinin or auxin was found more effective than using it individually [32]. The highest shoot multiplication ability of BA has also been reported for other *Silene* species; where MS medium supplemented with 4.48 μM BA was optimum for the multiplication of non-vitreous shoots of *Silene fabaria* with sufficient length [10]. Additionally, BA has been the most widely used cytokinin for improving the number and length of shoots in various *Silene* species; such as for regenerated shoots from callus of *Silene vulgaris* [32, 33] and axillary shoots from direct organogenesis in *Silene bolanthoides* [9].

Auxins are important PGRs for in vitro culture systems for root formation [10]. The natural IBA and the synthetic NAA auxins were chosen for rooting, because of their low oxidative rate and high stability in the plant in vitro culture. In particular, IBA is more stable than NAA; therefore, it is the most widely used auxin for root induction [10, 34]. The optimum medium for rooting of axillary shoots of *Silene schimperiana* was quarter-strength MS medium supplemented with 4.92 μM IBA in combination with 10.75 μM NAA. It gave 100% rooting with the highest mean number and length of roots per explant and mean length of axillary shoots. The number of roots is considered an important factor for enhancing the survival of plants during acclimatization and is a sign of a qualitative rooting response [35]. Concerning other *Silene* species, the present study gave rooting response on completely different PGRs combinations. For example; for *Silene leucophylla*, MS medium supplemented with 4 mg/L (17.94 μM) BA, 0.4 mg/L (2.16 μM) NAA, 0.2 mg/L (0.58 μM) GA_3_, 20% adenine sulfate, 0.05% silver nitrate, and 0.05% casein favored rooting of the proliferated shoots [6]. The optimal medium for rooting of *Silene fetissovii*, *Silene obovata*, *Silene sussamyrica*, and *Silene ladyginae* was MS medium supplemented with the addition of 1 mg/L (5.72 μM) indole acetic acid (IAA) [7]. In *Silene fabaria* subsp. *domokina*, 100% rooting was obtained by incorporating 0.1 mg/L (0.49 μM) IBA into the MS medium, followed by Kin and auxins combination [10]. On the other hand, in *Silene bolanthoides*, regenerated shoots were rooted on PGRs-free MS medium [9].

In the present study, a preliminary rooting experiment was carried out using each auxin individually and some of the treatments used with other *Silene* species from the literature (such as Saker et al. [6]. This experiment resulted in the production of weak unfunctional roots with a low percentage of rooting. This confirms that each plant species within a certain genus has its specific requirements and response to the propagation in vitro, which differs from the other species. Dolcet-Sanjuan et al. [34] confirm that the rooting response depends on different factors, such as type and concentration of auxin, species, and even the clone of a specific species. The plantlets with functional roots were successfully acclimatized in the greenhouse.

### Identification of *Silene schimperiana* by DNA barcode analysis

To identify and classify plant species for conservation, traditional taxonomic tools are inadequate. Recently, the alternative approach of DNA barcoding is successfully introduced for authentication of rare and endemic plant species as an important base for evolutionary and ecological studies as well as for determining conservation priorities [11, 13]. DNA barcoding provides proper identification of endemic plant species, which is very important to help in the conservation of natural plant genetic resources [20]. It is a reliable tool to identify a plant species and a short genetic sequence from a standard part of the genome that can be sufficient.

BLAST matching and phylogenic tree analysis of *Silene schimperiana* using the three markers: *ITS*, *rpoC1*, and *rbcL* as barcodes revealed 100% the genus *Silene*. It was reported earlier that on the species level, the identification of species is considered successful when the similarity percentage scores more than 95% and includes a single species. However, on the generic level, DNA barcoding is considered successful when all BLAST searches score similarity percentage of more than 95% and include a single genus [20]. In the present study, the highest similarity percentages between *Silene schimperiana* and the other recorded *Silene* species ranged between 99.03 and 99.38*.* The *rbcL* sequence showed 98.21% similarity with the same species; *Silene schimperiana* (unpublished data). Therefore, according to the obtained data, the identification of *Silene schimperiana* was successful on both species and genus levels for the *rbcL* marker and the genus level for *ITS* and *rpoC1* markers*.*

The phylogenetic trees of *Silene schimperiana* using the three markers: *ITS*, *rpoC1*, and *rbcL* and combined phylogenetic analysis supported the inclusion of *Silene schimperiana* in the genus *Silene* and the most closely related species are *Silene caesarea*, *Silene schimperiana*, and *Silene akinfievii.*

Phylogenetic analyses with a combination of nrDNA and cpDNA are one of the most effective methods to understand evolutionary relationships between and within species [36]. The results of the present study show a successful identification of *Silene schimperiana* on the species and genus levels. The success of species identification using DNA barcoding is contributing to the availability of nucleotide data of the corresponding taxa in the DNA sequences database [37]. However, additional experiments with other markers to entirely identify the plant more accurately are required. The present study reflects the potential use of DNA barcode analysis in documenting endemic endangered species concerning assigning to the proper taxonomic position.

## Conclusion

Biotechnology tools including in vitro propagation and DNA barcoding should be applied to rare and endemic plant species against the loss of wild plant populations and for germplasm conservation. Ex situ conservation by in vitro propagation provides a high yield for commercial utilization of valuable plant species, can be used for improving genetic characteristics and producing clonal plants [10]. DNA barcoding should be applied parallel to the morphological classification of plants for the identification of species and genetic relationships for appropriate conservation of plants [33].

**Fig. 8:**
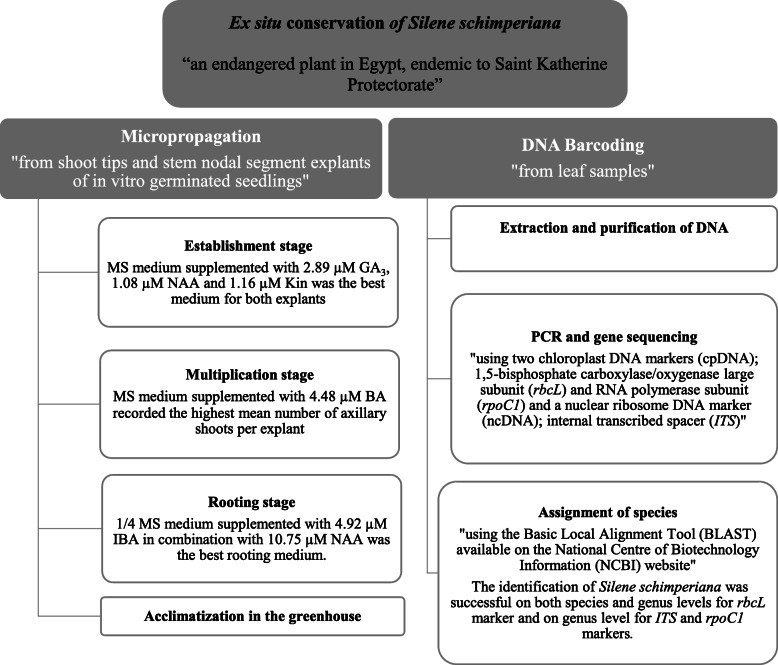
A schematic diagram of the ex situ conservation program done in the present study for *Silene schimperiana*

In conclusion, to the best of our knowledge, this is the first report on an efficient in vitro propagation protocol from shoot tip and stem nodal segment explants of *Silene schimperiana* with a satisfactory frequency of plant multiplication and production. Also, the molecular identification of *Silene schimperiana* was done using DNA barcode analysis for the proper conservation of the plant and to protect our intellectual property rights. A schematic diagram of the ex situ conservation program done in the present study for *Silene schimperiana* is presented in Fig. 8.

## Data Availability

All data generated or analyzed during this study are included in this published article.

## Abbreviations

ANOVA: Analysis of variance
BA: 6-benzyl adenine
BLAST: Basic local alignment tool
CBOL: Consortium for the barcode of life
cpDNA: Chloroplast DNA
GA_3_: Gibberellic acid
IBA: Iindole-3-butyric acid
*ITS*: Internal transcribed spacer
2iP: N6-(2-isopentenyl) adenine
IUCN: International Union for Conservation of Nature
Kin: kinetin
MS: Murashige and Skoog
NAA: α--naphthaleneacetic acid
NCBI: National Center of Biotechnology Information
ncDNA: nuclear DNA
PGRs: Plant growth regulators
*rbcL*: ribulose-1,5-bisphosphate carboxylase/oxygenase large subunit
*rpoC1*: RNA polymerase C1
TDZ: thidiazuron
